# Four weeks of augmented eccentric loading using a novel leg press device improved leg strength in well-trained athletes and professional sprint track cyclists

**DOI:** 10.1371/journal.pone.0236663

**Published:** 2020-07-29

**Authors:** Mellissa Harden, Alex Wolf, Martin Evans, Kirsty Marie Hicks, Kevin Thomas, Glyn Howatson

**Affiliations:** 1 Department of Sport, Exercise and Rehabilitation, Northumbria University, Newcastle, United Kingdom; 2 Directorate of Psychology and Sport, University of Salford, Greater Manchester, United Kingdom; 3 English Institute of Sport, Manchester, United Kingdom; 4 The Football Association, Burton on Trent, United Kingdom; 5 Water Research Group, North West University, Potchefstroom, South Africa; University of Rome, ITALY

## Abstract

This study assessed the efficacy of strength training using augmented eccentric loading to provoke increases in leg strength in well-trained athletes, and sprint track cyclists, using a novel leg press device. Twelve well-trained athletes were randomly allocated traditional resistance training (TRAD, n = 6), or resistance training using augmented eccentric loading (AEL, n = 6). A further 5 full-time, professional sprint track cyclists from a senior national squad programme also trained with augmented eccentric loading (AEL-ATH) alongside their usual sport-specific training. Participants completed four weeks of twice-weekly resistance training using the leg press exercise. In TRAD the lowering phase of the lift was set relative to concentric strength. In AEL and AEL-ATH the lowering phase was individualised to eccentric strength. Concentric, eccentric, isometric and coupled eccentric-concentric leg press strength, and back squat 1 repetition maximum (1RM), were assessed pre- and post-training. The AEL and AEL-ATH groups performed the eccentric phase with an average 26 ± 4% greater load across the programme. All groups experienced increases in concentric (5%, 7% and 3% for TRAD, AEL & AEL-ATH respectively), eccentric (7%, 11% and 6% for TRAD, AEL & AEL-ATH respectively), and squat 1RM (all p < 0.05), where the AEL-ATH group experienced relatively greater increases (13% vs. 5% in TRAD and AEL, p < 0.01). The TRAD and AEL groups also increased isometric strength (p < 0.05). A four-week period of augmented eccentric loading increased leg strength in well-trained athletes and track cyclists. The eccentric leg press stimulus was well-tolerated, supporting the inclusion of such training in the preparation programmes of athletes.

## Introduction

Muscular strength is a major contributing factor to athletic performance [1]. Greater muscular strength is associated with enhanced movement performance [2] and a decreased risk of injury [3] and as such appropriate resistance training to increase strength qualities is a cornerstone of athletic preparation programmes across a wide range of sports [4]. Conventional resistance training exercises, such as the squat and deadlift, are efficacious in improving muscular strength, however they are limited by the amount of mass the athlete is able to lift in the concentric phase. Conversely, humans are able to produce greater magnitudes of force during eccentric movements [5], and training strategies that afford an overload of eccentric muscle actions are potentially more efficacious than traditional resistance training [6–8], particularly for athletes with a long training history who might be limited in their potential to adapt to traditional resistance training methods [9–11]. The potential novelty offered by eccentric training strategies, coupled with the potential to elicit higher muscular forces than traditional training, makes such approaches attractive to well-trained athletic populations.

The application of high-intensity eccentric training is efficacious at improving strength, likely to a greater extent than concentric training as first demonstrated by Bradenburg & Docherty [12], however few studies have adopted an ecologically valid training approach. Following habitual use of high-intensity eccentric exercise there is evidence of increased maximum force producing capacity during eccentric, concentric and isometric exertions [6, 13], and numerous studies support the superiority of eccentric vs concentric training in eliciting improvements in measures of strength [6, 8, 13–15]. The majority of these studies employed isokinetic and/or single joint eccentric exercise, whereas in practice athletes typically perform multi-joint, compound movements. Two studies have demonstrated the superiority of eccentric resistance training regimes utilising augmented eccentric loading (AEL, where the load for the eccentric phase is >100% of concentric strength) for increasing strength in compound movements with well-trained athletes [16, 17]. Compared to traditional training, Cook *et al*. [16] observed greater improvements in upper and lower body strength, and vertical jump performance, and Douglas *et al*. [17] reported greater increases in lower body strength and sprint speed, after eccentric training. Furthermore, Coratella & Schena [11] showed that the improvements in strength elicited by AEL are maintained after a period of detraining, whereas those from traditional resistance training were not. These data indicate that the development of ecologically valid training regimes utilising high-intensity eccentric muscle actions could be more efficacious than traditional “concentric limited” resistance training in provoking adaptation to a range of athletic performance measures.

Although promising in application, there are significant logistical challenges associated with overloading the eccentric phase of resistance training movements. To this end, we have developed a novel leg press device capable of overloading eccentric muscle action in a lower body, bilateral, multi-joint movement [18, 19]. Our previous work has established the reliability of this stimulus [18], and its mechanical characteristics [19], as a foundation on which to prescribe training. The features of the device also allow the measurement of maximal concentric and eccentric strength, and thus training can be specifically prescribed relative to muscle action type, rather than prescribed to concentric strength [16, 17]. Such an approach is beneficial, particularly in highly strength trained athletes, in order to ensure task-specificity and account for potential individual differences in tolerance to eccentric exercise. The aim of this study was to ascertain the feasibility and efficacy of strength training with a novel leg press device that affords an overload of the eccentric phase on muscle function in well strength-trained individuals and in a group of professional sprint track cyclists when incorporating this approach to strength training alongside sport-specific training. Study of a group of professional, full-time, sprint track cyclists enabled us to quantify the feasibility and efficacy of a novel AEL stimulus in a group of highly-trained, professional athletes.

## Methods

### Design

A randomised, positive control trial was employed to test the efficacy of eccentric leg press training. Seventeen participants were recruited and allocated to 3 groups to complete traditional resistance training (TRAD, who acted as an active control group) [20] or augmented eccentric loading (AEL and AEL-ATH) performed on a bespoke incline leg press twice per week, for four weeks [18]. All groups performed leg-press exercise using a coupled eccentric-concentric movement with a five seconds tempo for the eccentric phase, and with maximum intent throughout the concentric phase. The difference between the groups was in the loading parameters for the eccentric phase; the TRAD group performed the descending phase with the same load as the ascending phase, which was prescribed based on concentric-only strength; the AEL and AEL-ATH groups performed the descending phase at an intensity relative to their eccentric-strength, and the ascending phase relative to their concentric strength, thereby offering an overload of the eccentric movement. The AEL-ATH group comprised full-time professional sprint track cyclists (AEL-ATH) from the Team GB senior academy programme (one level below the senior Olympic programme), to allow an assessment of the feasibility of the training stimulus in a professional, near-elite population. The training was preceded by two separate assessment sessions to measure individual strength profiles in back squat (day 1) and leg press exercise (day 2), The first pre-test session included familiarisation to the leg press machine and the tempo required for the eccentric phase. Participants were already habituated to traditional leg press and squat exercise from their own training. The two pre-test sessions were separated by 3 days. The two assessment sessions were repeated post-training separated by 3 days, after a 7-day deload. During the post-session testing period, the AEL-ATH group were subject to an unplanned, acute increase in their training load prior to their second assessment day, which meant one strength test could not be completed because of residual fatigue (traditional 1 repetition maximum leg press assessment, described below).

### Participants

Seventeen participants, twelve males and five females, gave written informed consent to participate in the study, which was approved by the Northumbria University Faculty of Health & Life Sciences Ethics Committee. Participant characteristics are presented in Table 1. Twelve participants were from a strength-power sport background (weightlifting, rugby, athletics, gymnastics, and combat), with 3–10 years of heavy resistance training experience; they were matched for 3 repetition maximum squat strength relative to body mass (3RM), and randomly allocated into two groups (AEL (n = 6; 1 female and 5 male) and TRAD (n = 6; 2 female and 4 male). The participants in the TRAD and AEL group were strength-trained, but not full-time professional athletes. The participants in the AEL-ATH group were full-time professional track sprint cycling athletes (n = 5; 2 female and 3 male). For the duration of the study, the AEL and TRAD groups were asked to avoid any lower body resistance training activity outside of the prescribed exercise programme and avoid unaccustomed resistance and cardiovascular exercise throughout the study duration. The participants in the AEL and TRAD groups continued to practice their sport two to three times per week at a moderate intensity and confirmed that they were not scheduled to compete or perform at high or maximal intensity within the study period. The AEL-ATH group continued with their on-bike track sprint cycling full-time training programme.

**Table 1:** Participant characteristics at baseline for each training group.

Group	*n*	Age (Yrs)			Stature (cm)			Mass (kg)		
AEL-ATH	5	19	±	0	174	±	13	76	±	12
AEL	6	28	±	2	179	±	7	82	±	9
TRAD	6	26	±	5	177	±	7	77	±	9

Values are mean ± SD.

### Procedures

#### Strength profiling

Strength was measured in leg press exercise (isometric, concentric, eccentric), and with a traditional back squat three repetition maximum. The bespoke leg press has been previously described [18, 19]. Briefly, the machine offers an overload of eccentric function via pneumatic technology, which can be immediately “unloaded” for the concentric phase of movement via adjustable magnetically operated reed switches. Force was measured via 4 s-type load cells (300 kg limit per cell) mounted onto the foot plates, which fed into a combinator to create a single voltage output. Associated with each load cell was a potentiometer (Hybritron®, 3541H1-102-L, Bourns, Mexico). The load cells and potentiometers sampled at 200 Hz. The voltage from the load cells and potentiometers were relayed into data acquisition software (LabVIEW 6.1 with NI-DAQ 6.9.2, National Instruments Corporation, USA) on a desktop PC. Force-time traces for each force plate (left and right) and displacement- and velocity-time traces for each potentiometer (left and right) were displayed. Raw data was exported from the data acquisition software into Microsoft Excel format (Microsoft Excel, 2010) and were analyzed offline.

*Isometric force assessment (ISO*_*90*_*)*. To determine maximum isometric force output, the leg press foot carriage was secured to ensure the required knee joint angle (90°, verified by goniometry). The 90° joint angle was chosen as it is commonly used for isometric assessment [21, 22]. Additionally, the 90° angle reflects the angle at the end range of motion (ROM) common to coupled eccentric-concentric exercise. Ratchet straps (>600 kg limit) were used to fix the carriage firmly in place to prevent unwanted movement and to maintain the integrity of knee and hip joint angle. Two preparatory efforts were performed at 50% and 75% perceived effort, separated by 30 seconds. Testing consisted of three 5 s maximal efforts interspersed by three minutes. During each effort, participants were instructed to ‘progressively build up force towards pushing as hard as possible until instructed to stop’. The same strong verbal encouragement was provided for all efforts. Unilateral force measures were summed to reflect the bilateral nature of the exercise. The trial with the highest peak force was used for analysis. Reproducibility has been established previously [18]; intraclass correlation coefficient (ICC): 0.92; coefficient of variation (CV: 3.4%); smallest worthwhile change (SWC): 3.3%.

*Traditional repetition maximum assessment (TRAD*_*1RM*_*)*. This assessment determined the maximum weight that could be moved through an initial lowering (eccentric) then lifting (concentric) phase to the nearest 5 kg for a single repetition. This was established within five attempts, separated by five minutes. The range of motion (ROM) was standardised to 90° of knee flexion. If full ROM was not achieved, then the effort was deemed a failed repetition. The maximum load lifted was recorded for analysis. Reproducibility of the measurement has been established previously; ICC: 0.98; CV: 2.2%; SWC: 3.25%.

*Maximum concentric force assessment (CON*_*1RM*_*)*. This assessment established the maximum force that could be produced during a concentric only movement. Participants performed efforts from a “dead” push at a knee joint angle of 90° with 5 minutes separating maximum attempts. The load was adjusted in 5 kg increments, and all participants achieved their maximum within 5 attempts. Training intensities for the concentric phase were based on CON_1RM_. The maximum force recorded during participants heaviest load lifted was recorded for analysis.

*Maximum eccentric force assessment (ECC*_*1RM*_*)*. This assessment determined the maximum force that could be imposed on the participant which could be controlled throughout the ROM of the descending phase of the leg press exercise for a duration of five seconds. The concentric phase was loaded with 50% of TRAD_1RM_. To standardise the pace of the eccentric phase, a custom-built LED strip with individually addressable LEDs (WS2812, BTF Lighting Technology Co. Ltd) controlled by a development board (Elegoo Mega 2560 R3, Elegoo Inc. UK & Arduino 1.8.4) and custom written code was added to the instrument. The LEDs lit up in a gradual manner to create a light trail that the participant followed, using a marker secured to the foot carriage. The length of the light trail (total number of LED lights) was pre-set to a distance that reflected the displacement of the foot carriage to a knee angle of 90° angle. The first eccentric effort was performed with a load which was equivalent to TRAD_1RM_. Load was increased by 5% until the five seconds pace set by the LED lights could no longer be maintained. Five minutes rest was prescribed between attempts. Following a failed effort subjects were given one further attempt at the load. In the event of a second failed attempt, force output associated with the preceding effort was used for analysis. Maximum force was recorded for analysis, which was achieved within six efforts for all participants. Reproducibility of the measurement has been established previously [18]; ICC: 0.93; CV: 3.0%; SWC: 2.9%.

*Squat three repetition maximum (SQ*_*3RM*_*)*. The maximum load that participants could complete 3, high bar back squat repetitions was recorded and used to prescribe subsequent training. The procedures to attain SQ_3RM_ followed a previously established protocol, yielding reproducible results (CV = 2.1%) for strength-trained individuals [23]. Participants squatted to full knee flexion, or where this was not possible to a depth where the femur was at least parallel to the floor. Pins were set in the squat rack corresponding to the barbell height achieved at the bottom of the squat to ensure consistent depth was achieved; this was visually confirmed by the lead investigator for each repetition. Participants lowered the load under control (approximately 3 s eccentric phase) and were instructed to immediately reverse the movement and perform the concentric phase as fast as possible. Participants completed sub-maximal warm-up repetitions until a load equating to 85% of predicted 1RM. Subsequently SQ_3RM_ was established in a maximum of five attempts, with five minutes rest permitted between efforts.

#### Training intervention

An overview of the training intervention is shown in Table 2. The intervention spanned seven weeks in total. Week one and week seven were allocated to baseline and post-testing, respectively. Week two through to week five comprised the training period. Week six was allocated to a period of deload. During the main training period, progressive overload was achieved through a gradual increase in intensity (%1RM determined relative to either ECC_1RM_ or CON_1RM_, as described below) each week, starting with a range between 82.5–87.5% 1RM in week one to 97.5–102.5% 1RM in week 4. To illustrate this progression, in week 1 for both leg press and back squat exercises, participants performed sets of 3 repetitions at 82.5%, 85% and 87.5% 1RM. In the final training week participants performed the same sets and exercises with loads of 97.5%, 100% and 102.5% of 1RM. Table 2 provides full details of the exercise intervention and programming variables.

**Table 2:** Overview of the training intervention. The AEL and AEL-ATH groups performed leg press exercise with an augmented eccentric (ECC) phase (the ECC intensity was set relative to maximum ECC strength). The TRAD group performed leg press exercise in a traditional manner where both concentric (CON) and ECC phases were prescribed to CON repetition maximum strength.

		**Training overview**						
**Week number**		1	2	3	4	5	6	7
**Objectives**		Familiarisation Strength assessment	Training 1	Training 2	Training 3	Training 4	Deload	Strength assessment
**Intensity classification**		Very heavy	Moderate	Moderate-Heavy	Heavy	Very Heavy	Moderate	Very Heavy
**Sessions per week**		2	2	2	2	2	2	2
		**Exercise prescription (Sets × Reps %1RM)**						
		*Leg press load was set relative to ECC_1RM_ for AEL and AEL-ATH, and relative to CON_1RM_ for TRAD						
**1**	Leg press*	Max	4×3 82.5–87.5%	4×3 87.5–92.5%	4×3 92.5–97.5%	4×3 97.5–102.5%	3×3 82.5%	Max
**2**	**Back squat**	Max	3×3 82.5–87.5%	3×3 87.5–92.5%	3×3 92.5–97.5%	3×3 97.5–102.5%	3×3 82.5–87.5%	Max
**3**	**Pull from floor**	N/A	3×6 70–75%	3×6 70–75%	3×6 70–75%	3×6 70–75%	3×3 70%	N/A
	**Conditioning circuit:**	N/A	3 rounds:	3 rounds:	3 rounds:	3 rounds:	N/A	N/A
**4a**	SL goblet squat		×8 reps	×8 reps	×8 reps	×8 reps		
**4b**	Isometric trunk hold		×30 s	×30 s	×30 s	×30 s		
**4c**	Lying leg raise		×10 reps	×10 reps	×10 reps	×10 reps		

Participants performed the same S&C programme, the only difference was in the load prescription for leg press exercise. The AEL and AEL-ATH groups performed coupled eccentric-concentric leg press exercise with load for the eccentric and concentric phase prescribed relative to ECC_1RM_ and CON_1RM_, respectively, thereby offering a precise overload of the eccentric phase. The TRAD group performed coupled eccentric-concentric leg press exercise with the same load during both phases, which was prescribed relative to CON_1RM_. The AEL-ATH group also continued their on-bike track sprint cycling training.

### Statistical analysis

Values are reported as mean ± SD. All data sets were checked for normality using Shapiro-Wilk’s test (*p ≤* 0.05). Programme characteristics were examined using a one-way ANOVA to determine the differences in training intensity between the TRAD, AEL and AEL-ATH groups. To assess the effect of training, mixed 2 × 3 ANOVAs with a within-subject factor of time (Pre- vs. Post-training) and between-subjects factor of group (TRAD vs. AEL vs. AEL-ATH) were performed to assess changes in strength diagnostics. The main ANOVA models included *η*_***p***_^*2*^ effect sizes, and significant main effects were followed by Least Significant Difference *post-hoc* tests. Relative changes from pre- to post-training are presented using forest plots displayed as $\bar{x}$ ± 95% CI, with an illustration of the measurement error of the test. Hedges g was used to quantify effect sizes, interpreted as small (>0.2), medium (>0.5) and large (>0.8) [24]. Statistical significance was accepted at *p* < 0.05.

## Results

### Programme characteristics

Relative training intensity performed during the concentric phase was not different between the three groups (F _(2, 21)_ = 2.3, *p =* 0.12, η_***p***_^2^ = 0.18). Relative training intensity performed during the eccentric phase was different between groups (F _(2, 21)_ = 24.5, *p <* 0.01, η_***p***_^2^ = 0.70), whereby TRAD trained with lower relative intensity compared to both AEL (−8.42 N^.^kg^-1^, −11.74 to −5.09 N^.^kg^-1^) and AEL-ATH (−6.80 N^.^kg^-1^, −10.12 to −3.48 N^.^kg^-1^). The AEL-ATH and AEL groups performed the eccentric phase with 26 ± 4% greater intensity across the training intervention compared to TRAD.

### Strength diagnostics

**Fig 1:**
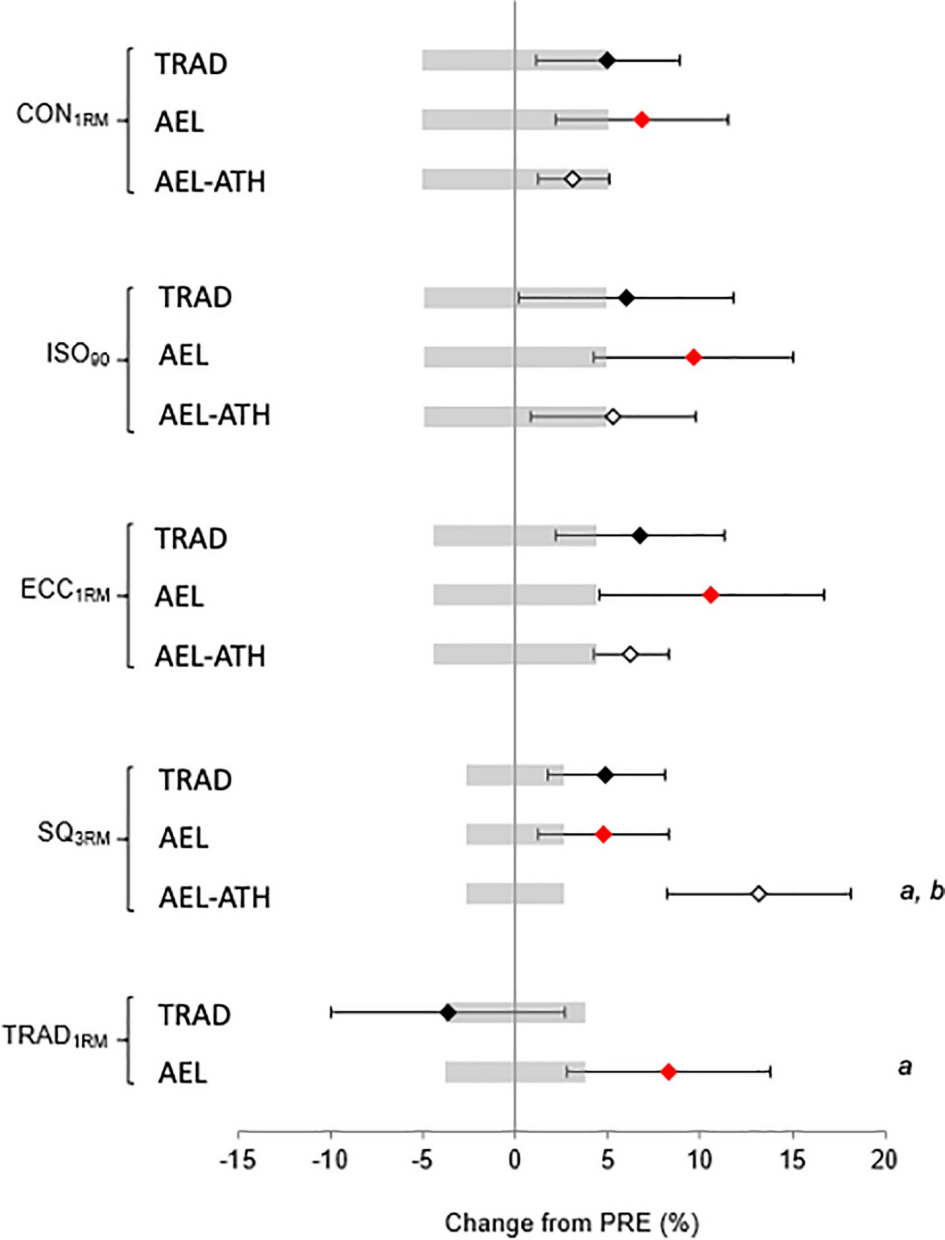
Relative changes (± 95% confidence intervals) in strength following training with (AEL, AEL-ATH) and without (TRAD) augmented eccentric loading. Shaded bars represent the measurement error for each outcome measure. *a*, different from TRAD, *b*, different from AEL (*p* < 0.05).

All training groups experienced increases in strength post-training. Eccentric repetition maximum increased for all groups (Table 3, Fig 1), with no difference between groups in the magnitude of change (group × time *p* = 0.25, Table 3), which equated to effect sizes of 0.7, 0.4, and 0.3 for AEL, TRAD, and AEL-ATH respectively. Both the TRAD and AEL groups improved ISO_90_ (AEL, *p* < 0.01, g = 0.7; TRAD, *p* = 0.04, g = 0.4), and CON_1RM_ (AEL, *p* < 0.01, g = 0.5; TRAD *p* = 0.02, g = 0.3) strength post-training. The change in ISO_90_ (1.8 N^.^kg^-1^, −0.1 to 3.7 N^.^kg^-1^, *p* = 0.06, g = 0.4) and CON_1RM_ (1.1 N^.^kg^-1^, −0.2 to 2.4 N^.^kg^-1^, *p* = 0.10, g = 0.2) for the AEL-ATH group did not attain statistical significance.

**Table 3:** ANOVA model statistics, and changes in strength diagnostics following training with (AEL, AEL-ATH) and without (TRAD) augmented eccentric loading. Significant pre- to post-training changes within-group are denoted with * (*p* < 0.05).

								ANOVA								
		PRE			POST			Group			Time			Group x Time		
Variable	Group	$\bar{x}$	±	SD	$\bar{x}$	±	SD	*F*	*p*	η_p_^2^	*F*	*p*	η_p_^2^	*F*	*p*	η_p_^2^
CON_1RM_ (N·kg^-1^)	TRAD	32.2	±	4.4	33.7	±	3.75*	1.49	0.26	0.18	22.60	< 0.01	0.62	0.91	0.43	0.12
	AEL	34.1	±	4.5	36.3	±	3.57*									
	AEL-ATH	36.8	±	5.0	37.9	±	4.60*									
ISO_90_ (N·kg^-1^)	TRAD	33.3	±	4.9	35.1	±	3.91*	0.88	0.44	0.11	22.45	< 0.01	0.62	0.91	0.43	0.12
	AEL	34.5	±	4.6	37.6	±	3.37*									
	AEL-ATH	36.8	±	5.2	38.6	±	4.74									
ECC_1RM_ (N·kg^-1^)	TRAD	35.7	±	5.5	38.0	±	5.13*	2.15	0.15	0.24	38.93	< 0.01	0.74	1.56	0.25	0.18
	AEL	40.4	±	5.2	44.6	±	5.40*									
	AEL-ATH	42.2	±	7.1	44.8	±	7.36*									
TRAD_1RM_ (N·kg^-1^)	TRAD	34.6	±	5.3	33.4	±	5.50	2.42	0.15	0.20	1.05	0.33	0.10	7.36	0.02	0.42
	AEL	36.5	±	3.6	39.4	±	3.53*									
	AEL-ATH															
SQ_3RM_ (kg·BW^-1^)	TRAD	1.59	±	0.27	1.66	±	0.25*	0.54	0.60	0.07	61.23	< 0.01	0.81	7.47	0.01	0.52
	AEL	1.70	±	0.19	1.78	±	0.14*									
	AEL-ATH	1.62	±	0.18	1.83	±	0.15*									

CON_1RM_, leg press concentric maximum force; ISO_90_, leg press isometric maximum force at 90° knee angle; ECC_1RM_, leg press eccentric maximum force; TRAD_1RM_, coupled eccentric-concentric leg press maximum force; SQ_3RM_, three repetition maximum back squat relative to body mass.

For SQ_3RM_ and leg press TRAD_1RM_ there were significant group × time interactions. Specifically the AEL-ATH group increased SQ_3RM_ to a greater extent (g = 1.1) than both AEL (p < 0.01, g = 0.4) and TRAD (p = 0.01, g = 0.3) (Table 3, Fig 1). For leg press TRAD_1RM_ there was no main effect for time (p = 0.33) however the increase in the AEL group (2.93 N^.^kg^-1^, 0.46 to 5.40 N^.^kg^-1^, g = 0.8) was different to the slight decrease in the TRAD group (−1.32 N^.^kg^-1^, −3.80 to 1.15 N^.^kg^-1^, g = −0.2, group × time p = 0.02). The AEL-ATH group did not perform leg press TRAD_1RM_ post-testing due to the aforementioned, unexpected, residual fatigue.

## Discussion

The aim of this work was to ascertain the feasibility and efficacy of training with a novel leg press device, that affords an overload of muscle lengthening actions, in well-trained strength athletes and professional sprint track cyclists. Four weeks of eccentric strength training provoked improvements in a range of strength diagnostics, with some evidence of greater magnitudes of improvement in comparison to traditional strength training. In a group of professional cyclists, a four-week eccentric training stimulus was successfully implemented, and efficacious at improving measures of muscular strength, including a relatively greater increase in 1RM squat strength. Thus, a short-term (4 weeks, 8 sessions) eccentric strength training programme was efficacious, and well-tolerated by well-trained strength athletes and sprint track cyclists, and offers a novel training exercise to improve indices of muscle strength.

The four-week training programme employed was sufficient to provoke improvements in a range of strength characteristics, with some specific differences between groups in training-induced changes in strength. All groups improved concentric, eccentric and isometric leg press strength, and the magnitudes of improvement were comparable to those previously reported for strength-trained individuals for short term (4 to 6 weeks) training [11, 17, 25], but less than that observed for longer-term (10–12 weeks) training incorporating augmented eccentric loading [16, 26]. The AEL group improved leg press 1 repetition maximum to a greater extent than the TRAD group, and the AEL-ATH group improved their squat 3 repetition maximum more than both TRAD and AEL. With respect to the AEL group, the greater increase in leg press 1RM compared to TRAD suggests a possible greater efficacy of training with augmented eccentric load. This is partially supported by a relatively greater change in concentric (7% vs. 5%), eccentric (11% vs. 7%) and isometric (10% vs. 6%) strength in AEL compared to TRAD, however these interpretations should be treated with caution as none of these comparisons attained statistical significance. The training programme employed was relatively short (4 weeks, 8 sessions), and it is plausible to speculate that a longer duration might have revealed further differences between groups. This notion is supported by the findings presented by Walker *et al*. [26] which highlight that the benefits of augmented eccentric loading for strength-trained individuals might take some time to manifest (more than five weeks). Thus, future research that builds on this preliminary data should consider implementing a longer training period.

The AEL-ATH group incorporated the eccentric overload stimulus alongside their usual S&C programme, and track cycling training. In this group there were large changes in squat repetition maximum strength that exceeded the magnitude of change observed in TRAD and AEL, and was greater than that previously reported in athletic cohorts [16, 17]. This was in contrast however to the relatively modest changes in concentric, eccentric and isometric strength. This can be explained in part by an unexpected interruption to the athletes training schedule during the post-testing period. Testing of squat repetition maximum was performed on post-testing day 1 of 2, after a suitable deload period. However, on the second day of testing, which comprised leg press strength diagnostics, the AEL-ATH group were experiencing residual fatigue as a consequence of an unexpectedly high acute training load, and the preceding strength assessment. This also necessitated the termination of the testing procedures before the traditional leg press 1RM was assessed. The requirements for the organisation of post-testing and time allocated for recovery needed to be adapted for the AEL-ATH group to account for the logistical constraints surrounding full-time professional sport participation. These factors were beyond the experimenter’s control, and as such the true impact of the training programme might not have been revealed as a consequence, though it is worth noting that both concentric and eccentric strength scores were higher, despite this residual fatigue compromising the testing schedule. This notwithstanding, the present study does demonstrate that strength training exercises overloading eccentric muscle actions can be feasibly incorporated into the training programme of full-time professional sprint track athletes, and could potentially provoke greater improvements in strength compared to traditional training.

A relatively novel feature of the study was the utilisation of a progressive loading approach based on muscle action specific 1RM for the eccentric and concentric phase across the training period. The training intensity experienced by the AEL and AEL-ATH groups was 23–30% higher during the leg press exercise, because of the progressive overload of the eccentric phase of the exercise. Previous work has typically programmed load for the eccentric phase relative to concentric strength [16, 17, 25, 26], whereas the approach adopted here allowed for the exploitation of the greater force producing capacity associated with eccentric muscle actions [5, 6, 8], whilst accounting for individual differences in eccentric strength. The optimal prescription of augmented eccentric load is unknown, but this range could provide a suitable guideline for those practitioners that do not have access to equipment that facilitates the safe evaluation of eccentric-specific strength. A limitation of this approach is the inability to match volume between groups. Exercise volume is a key factor in resistance training prescription and, given the nature of the exercise prescription relative to their maximum eccentric force, the AEL group would have experienced a greater volume compared to TRAD. Coratella *et al*. [27] recently highlighted the importance of matching volume in resistance training studies. Though this wasn’t achieved in the present study, the approach taken here is arguably more ecologically valid, as typically practitioners using AEL would aim to exploit the higher force-producing capacity of eccentric muscle actions. Further research to uncover the mechanisms underpinning adaptation to different forms of training would benefit from a more controlled approach [27].

The results of this study support the efficacy of training with augmented eccentric loading for improving leg strength qualities in well-trained athletes. A further important practical application is the palatability of the training stimulus in a full-time, professional athletic population. The AEL-ATH group successfully incorporated the eccentric training stimulus alongside their usual sport-specific training programme, with no adverse outcomes reported. Anecdotally, a number of riders also verbally reported increased feelings of stability when returning to their usual compound lifts, which was reflected in the large improvements demonstrated in squat strength. A limitation of the current work is the relatively small sample sizes and short duration of the study have hindered any conclusions on the relative superiority of augmented eccentric loading, and the logistical constraints imposed by testing a professional population rendered more sport-specific assessment unfeasible. Furthermore, the similarity of the testing modality (leg press strength in isometric, concentric, eccentric, and traditional modes) to the training stimulus, and the known specificity of adaptation to eccentric loading [28] also limits the generalisability of the results to other movements. Further work is warranted to ascertain whether the improved strength offered by AEL using leg press exercise leads to improvements in other, sport-specific movements. Nonetheless, the results demonstrate that training with augmented eccentric loading is efficacious at improving leg strength in well-trained athletes, and can feasibly be incorporated into a professional, full-time athlete programme with no adverse consequences.

The approach to exercise prescription in this study was high in ecological validity; loads were prescribed in an attempt to optimise the stress of the AEL stimulus at the expense of matching for volume load, and the leg press exercise was incorporated into the wider resistance training program and was thus executed alongside other exercises. While this approach reflects what would occur in practice, it limits the ability to attribute causation given that the leg press stimulus was not the sole strength exercise the groups were performing. In an attempt to isolate the effect of the leg press stimulus, the additional training performed by all groups was matched for volume, intensity, and muscle action type. As a consequence, the only difference between groups was in the prescription of leg press exercise; this increases our confidence that the observed differences between groups could be due to the differing exercise prescription, however it does not allow us to isolate the effect of the leg press exercise *per se*.

A final potential limitation of the study is that the AEL-ATH group were younger than the AEL and TRAD groups. The AEL-ATH group were full-time, professional track cyclists who, though young in chronological age, were experienced with resistance training (>2 years), and were exhibiting levels of leg strength that were not different to the other groups. Anecdotally, the athletes in this group were reaching a plateau in their response to resistance training and the load required to achieve overload in traditional compound movements was becoming so high as to negatively impact their sport-specific training. However, given the younger age of this group, it is not possible to discount the possibility that they had a relatively greater capacity for adaptation, and thus the greater increase in squat strength observed in the AEL-ATH group could be partly attributed to their younger training age.

In conclusion, four weeks of resistance training that incorporated augmented eccentric load, imposed via a novel leg press device, provoked marked adaptation in a range of muscle strength qualities, with evidence of a greater magnitude of improvements compared to traditional resistance training. The eccentric training stimulus imposed a greater demand compared to traditional resistance training, but this was well-tolerated by all participants, including a group of sprint track cyclists. This indicates that training with augmented eccentric load is both feasible, and efficacious for athletes aiming to improve muscle strength qualities. Future research studying training over longer time periods is warranted to fully understand the longer-term consequences and adaptations to this type of training.

## Supporting information

S1 Data (XLSX)
